# Findings from the Promoting Independence in Dementia App (PRIDE-app) Study a Reach, Effectiveness, Adoption, Implementation, and Maintenance Framework Discussion

**DOI:** 10.1177/08919887241246237

**Published:** 2024-04-10

**Authors:** Abigail Rebecca Lee, Orii McDermott, Martin Orrell

**Affiliations:** 1 Department of Mental Health and Clinical Neurosciences, 170718 University of Nottingham School of Medicine, Nottingham, UK

**Keywords:** Dementia, psychosocial, quality of life, self-management, intervention, online, reach, effectiveness, adoption, implementation, and maintenance

## Abstract

**Introduction:**

Self-management is pivotal in helping people with their independence and in managing their health conditions more effectively. The PRIDE-app is a novel online intervention, providing support and information for people living with dementia and their families, aimed at increasing self-management and improving quality of life. Knowledge generated will help inform future developments to the app, with the aim of improving its uptake and implementation in services.

**Methods:**

A mixed-methods approach incorporating the RE-AIM framework. Recruited 25 people living with dementia, of which 17completed the PRIDE-app intervention over 8 weeks with support from a dementia adviser facilitator. Measures exploring mood, physical well-being, and quality of life were collected at baseline, 3 and 6 months and analysed through modelled analysis. Post-intervention interviews were conducted with participants and facilitators and analysed through thematic analysis.

**Results:**

Quantitative results did not show significant improvements in participants’ scores. Qualitative data showed that the PRIDE-app motivated people to reconnect socially and set individual goals for activities. Participants and facilitators identified areas for improvements to the app interface and delivery format.

**Conclusions:**

This study evaluated the PRIDE-app’s reach, effectiveness and adoptability in the independence and quality of life of people living with dementia, as well as how it could be implemented and maintained within services. Pre- and post-intervention scores were inconclusive. Interviews provided positive feedback of the app’s influence on peoples’ activities and mood.

## Introduction

Self-management in dementia has potential for helping people and their families to retain control over their lives. Mountain^1^ highlighted the integral role that physical and mental wellbeing plays in successful self-management. Psychosocial interventions are one approach to implementing successful self-management, as they have the potential to enable people living with dementia to have a better quality of life.^2^

The Promoting Independence in Dementia (PRIDE) program was developed with the goal of supporting people living with mild dementia living in the community who wish to maintain their independence despite progression of their dementia.^3^ The program incorporates self-selected mental, physical and emotional topics and activities, rather than just targeting one aspect such as memory or mood as many dementia-specific interventions do.^4^ PRIDE is presented in a manual and incorporates several principles of self-management that promote positive lifestyle changes and the importance of social inclusion.^3^ There is the opportunity for an informal supporter, such as a relative or friend, to be involved with PRIDE, but the program is primarily aimed at people living with mild dementia. To help guide the activity plans and promote positive behaviour change, PRIDE incorporates 3 steps: planning, doing, and reviewing.^5^ The first step is to select an activity or action, then to consider what would encourage that activity or action and any practical factors that would facilitate or prevent it. The last step is there to encourage reflection and the application of problem-solving strategies to alter, refine or strengthen the activity plan.^5^ Users work collaboratively with a Dementia Advisor (DA), who facilitates the intervention sessions and promotes personalized activity plans.

The RE-AIM (Reach, Effectiveness, Adoption, Implementation, and Maintenance) framework^6^ enabled us to identify key components for effective adoption, successful implementation, and sustained use of the PRIDE-app, and identify potential barriers to the wider use of web-based psychosocial interventions for dementia.

## Methods

### Study Design

We conducted a pre-post feasibility study of the PRIDE-app in people living with mild dementia in the United Kingdom between June 2021 and September 2022. Given the barriers of conducting research during the COVID-19 pandemic, we conducted all stages of the project remotely, including site recruitment and set up, training of facilitators, and participant interviews. The PRIDE-app intervention sessions were also held remotely via video or telephone. Our recruitment target also had to be lowered from 250 to 60, as sources of in-person recruitment we initially planned on using, such as memory clinics, were no longer viable. The project was reviewed and approved by the Oxford Research Ethics Committee (21/SC/0066), and all participants, supporters and facilitators provided written informed consent before taking part. Each site scanned copies of signed consent forms and emailed them securely to the research team. The full method for this study is outlined in our published protocol.^7^

Outcomes were evaluated through the RE-AIM framework,^6^ with quantitative data gathered through participant demographics, retention figures and pre- and post-intervention scores on the Control, Autonomy, Self-realization, and Pleasure Scale-19,^8^ Lawton Instrumental Activities of Daily Living Scale,^9^ EQ-5D-5L,^10^ Geriatric Depression Scale,^11^ Engagement and Independence in Dementia Questionnaire,^12^ and Global Change Measure.^13^ Supporters completed the General Health Questionnaire,^14^ EQ-5D-5L, and a proxy-rated Global Change Measure Qualitative data was assessed through interviews with participants, supporters, and facilitators.

### Inclusion Criteria

To participate in the study, participants had to be aged 18 years or over; have a medically confirmed diagnosis of dementia; able to provide informed consent and engage with the intervention; and have access to Wi-Fi, a computer or tablet computer, telephone number and email address.

### Interview Questions

The aim of the interviews was to explore the perspectives and experiences not covered in the quantitative outcome measures. The lead researcher (ARL) and a second researcher (OM) developed questions for people with dementia and their supporters that explored their experiences of the app. The final semi-structured schedule was developed with the involvement of a person with lived experience of dementia, with a similar schedule developed for DAs. All interviewees were given the freedom to expand on any points they voiced, and the final question on each schedule allowed for them to add anything that had not been discussed.

### Interview Sample

During the consent process, people with dementia, supporters and DAs indicated whether they would be interested in taking part in post-intervention interviews. Of the 17 participants, 13 consented to being contacted for interview. Of the 13 invites sent out, nine interviews were conducted with two participants with dementia, two supporters, one dyad, and three facilitators. Interviews were conducted on a one-to-one basis by ARL and OM over Microsoft Teams or by telephone and lasted between five and 50 minute. Verbal consent was obtained on the day, prior to recording the interview, to ensure participants still wished to take part. All interviewees were provided with a copy of their respective question schedule a week before their interview date.

### Intervention

During the feasibility trial of the PRIDE program, the research team at University College London transformed the content from a paper-based handbook to an online format called the PRIDE-app.^13^ Researchers at the University of Nottingham worked collaboratively with the app development company and target users to create a feasibility prototype. The PRIDE-app comprises three core topics: *Finding a Balance; People and Connections;* and *Keeping Going.* Users can select to focus on three of seven additional topics: *Keeping Mentally Active; Keeping Physically Active; Keeping Socially Active; Making Decisions; Getting Your Message Across; What Does it Mean to be Told You Have Dementia;* and *Keeping Healthy*.^5^ Woven through the content are stories about the difficulties other people living with dementia have faced, which aims to help promote discussions during and between sessions. PRIDE-app is delivered through three sessions with a DA, a trained facilitator who supports people with mild dementia and their family throughout the program.^5^ DAs and people with dementia work collaboratively through the manual, identifying activity plans and social participation needs, and they discuss resources available to support them. Participants and supporters trialled the PRIDE-app across 8 weeks, attending three facilitated sessions, and tried to implement the app techniques and information into their daily lives.

### Data Analysis

ARL transferred the interviews into audio files before sending them to an independent transcription company, dictate2us, where they were transcribed verbatim. Any identifying material was removed to ensure anonymity and each transcription was given an individual identification code (e.g., PP1, PP2). Reflexive thematic analysis was chosen as the method of analysis as its flexibility suited the overall RE-AIM framework and would enable a better understanding of the thoughts and experiences within the qualitative data.^15,16^ ARL followed the phases of reflexive thematic analysis outlined in Braun and Clarke^15^:

1. Familiarization – ARL and OM independently read through the interview transcripts several times, and ARL listened to the audio recordings again. Both reviewers made initial notes about the individual data items and the overall dataset.
2. Coding – Once well familiarized with the data, ARL and OM worked through the transcripts independently to identify and code potentially relevant sections.
3. Generating initial themes – Shared meaningful ideas across the data set were identified. The coded data which made up these ideas were collated together to create initial themes. ARL and OM then discussed which sections they had identified and coded and why.
4. Developing and reviewing themes – Following the reviewers’ discussions, ARL created documents for each transcript to check that the themes made sense in relation to the coded data and the overall dataset. Revisions were made to initial themes to either expand them, split them into new themes, or discard them if they were not deemed to highlight the most important points of the dataset.
5. Refining and defining themes – ARL and OM then reviewed the revised themes and added additional independent thoughts about the reflections and interview experience.

## Results

### Quantitative Results

#### Recruitment Figures and Participant Demographics

The study was advertised through the Clinical Research Network (CRN) Portfolio, and local CRN teams were able to contact the lead researcher (ARL) to register their interest. A total of 19 sites requested more information about the study between February 2021 and January 2022, and four had the capacity and capability to support the study. Participant recruitment opened at sites and on the Join Dementia Research (JDR) online platform in June 2021 and ended in February 2022. Two of the four sites utilised JDR, contacting 16 interested volunteers and enrolling three. Overall, 28 people completed the participant baseline. However, on review of the data, three of these were uncompleted duplicates. Therefore, 25 people with a diagnosis of mild dementia completed the online baseline measures, of which 15 were recruited with a supporter, although only 12 completed the full set of baseline measures.

All participants and supporters were white British. One non-white person with dementia registered their interest via JDR, but they did not respond to the email invite to learn more about the study. 22 (80%) participants said they would like to take part with a family member or friend. However, only 15 of the nominated supporters responded to the questionnaire link sent to them. Of these supporters, 10 (83.3%) were a spouse or partner of the participant, and 2 (16.7%) were a son or daughter (Table 1). All interviewees were over the age of 18 years and consisted of six females and three males.

**Table 1.** Demographic Characteristics of participants who Completed Baseline Measures.

Demographic	Participant
Region	South West n = 17(68%)
	West Midlands n = 3(12%)
	South East n = 2(8%)
	North West n = 1(4%)
	Yorkshire n = 1(4%)
	Scotland n = 1(4%)
Marital Status	Married/Civil Partnership/Cohabiting n = 20(80%)
	Widowed n = 3(12%)
	Separated/Divorced n = 1(4%)
	Single n = 1(4%)
Living Arrangements	With a Spouse/Partner n = 19(76%)
	With Family n = 4(16%)
	Alone n = 2(8%)
Taking Dementia Medication	Yes n = 20(80%)
Current/Previous Smoker	Yes n = 10(40%)
Weekly Alcohol Consumption	1 day or Less n = 13(52%)
	5+ Days n = 5(20%)
Weekly Exercise	2 hours + n = 15(60%)
Prior Experience with Technology	None n = 7(28%)
	Some n = 11(44%)
	Quite a lot/Extensive n = 7(28%)

#### Participant Retention

Following the completion of baseline measures, invites were sent to all those participants and supporters who had consented. The teams received 19 responses, of which 17 completed one or more PRIDE-app sessions with their DA. Completion rate dropped off between timepoints due to the reduced number of participants who completed the intervention phase. At baseline, 25 people with dementia and 15 supporters completed the measures. These figures dropped at 3 months to 16 participants and five supporters, and also at 6 months with 15 people with dementia and seven supporters completing all outcome measures. One couple completed the follow-up measures but did not use the PRIDE-app.

#### Pre/Post Outcome Measure Performance

Due to the nature of the questionnaire, the GDS was examined separately to the modelled analysis completed on the other measures. A score of five or below on the GDS was classified as not depressed, compared to six or more being regarded as depressed. At baseline, 20 people with dementia showed no signs of depression and five did. The number of participants who completed the measure dropped at 3 and 6 months, but only two people with dementia showed signs of depression at both follow-ups. Most people with dementia reported no signs of depression at baseline or either of the follow-ups, and there was no significant difference across timepoints (Fisher Exact Test *P* = .811).

Table 2 details the modelled means and change in scores between baseline and follow-ups.

**Table 2.** Modelled Mean Scores and Change Significance Across Outcome Measures.

Measure	Month	Modelled Mean (95%CI)	Change (95%CI)	*P*-value
IADL^a^	Baseline	8.00 (7.42, 8.58)		
	3	8.00 (7.42, 8.58)	Month 3 v Baseline	N/A
			0	
	6	8.00 (7.42, 8.58)	Month 3 v Baseline	N/A
			0	
EID-Q^b^	Baseline	74.67 (68.96, 80.37)		
	3	72.34 (66.06, 78.62)	Month 3 v Baseline	.345
			−2.33 (−7.16, 2.51)	
	6	74.20 (67.83, 80.57)	Month 6 v Baseline	.853
			−.47 (−5.42, 4.49)	
CASP-19^c^	Baseline	42.46 (39.32, 45.60)		
	3	40.01 (36.40, 43.63)	Month 3 v Baseline	.157
			−2.44 (−5.83, .94)	
	6	40.89 (37.20, 44.58)	Month 6 v Baseline	.376
			−1.57 (−5.04, 1.90)	
EQ-5D-5L^d^	Baseline	70.74 (63.21,78.27)		
	3	65.79 (57.10, 74.48)	Month 3 v Baseline	.272
			−4.95 (−13.78, 3.88)	
	6	72.13 (63.23, 81.02)	Month 6 v Baseline	.764
			1.38 (−7.66, 10.43)	
EQ-5D-5L	Baseline – Supporter	86.75 (82.18, 91.32)		
	3 – Supporter	77.66 (70.79, 84.52)	Month 3 v Baseline	.007
			−9.09 (−15.75, −2.44)	
	6 – Supporter	80.19 (74.31, 86.07)	Month 6 v Baseline	.023
			−6.56 (−12.20, −.92)	
GHQ-12^e^	Baseline – Supporter	25.58 (23.03, 28.14)		
	3 – Supporter	19.17 (15.80, 22.54)	Month 3 v Baseline	.000
			−6.41 (−9.50, −3.33)	
	6 – Supporter	22.70 (19.68, 25.72)	Month 6 v Baseline	.036
			−2.88 (−5.58, −.19)	

^a^IADL: Lawton Instrumental Activities of Daily Living Scale.

^b^EID-Q: Engagement and Independence in Dementia Questionnaire.

^c^CASP-19: Control, Autonomy, Self-realization, and Pleasure Scale-19.

^d^EQ-5D-5L: EuroQoL Quality of Life.

^e^GHQ-12: General Health Questionnaire – 12.

#### Participants

##### Lawton IADL, EID-Q and CASP-19

All participants reported a maximum score of eight on the Lawton IADL measure, which would indicate a good level of functioning and independence, at baseline and both follow-ups. The maximum score that participants could achieve on the EID-Q measure was 104. The table shows that there was no difference between baseline and follow-ups, with a slight deterioration at 3 months compared to baseline, but this stabilized by 6 months. On the CASP-19, a maximum score of 57 is achievable with the Likert scoring method, with a higher score indicating a better level of wellbeing. As shown in the outcome data, there was no difference in participants’ wellbeing before and after using PRIDE-app.

##### EQ-5D-5L

The mean EQ-5D-5L visual health scores for participants and supporters, along with the modelled means, are shown in the table above. Modelled analysis between baseline and follow-ups showed no change in scores for participants living with dementia.

##### Global Change Measure

At 3 months, 75% (n = 12) of participants reported no change in their general wellbeing, one rated their wellbeing as much worse, and three participants felt that their wellbeing had improved slightly. With regards to their independence, participants felt there tended to be no change (75%, n = 12), although two felt they had become a bit more independent, and another two less independent.

At the 6-month follow-up, just over half (53.3%, n = 8) of participants reported no change in wellbeing since starting the PRIDE-app study, with 26.7% (n = 4) saying it was a bit worse, and one participant much worse. Many participants (73.3%, n = 11) reported no change in their independence in the 6 months since the study started. One participant felt much more independent and three felt less independent.

#### Supporters

##### EQ-5D-5L

The analysis showed a significant decline in supporter scores at 3 months (*P* < .01) and at 6 months (*P* < .05), indicating that they felt their quality of life had deteriorated.

##### GHQ-12

The Likert scoring method of 0-1-2-3 was chosen for analysis as it was felt this method would provide more insights into differences between supporters and timepoints than the 0-0-1-1 scale. Better reported health was indicated by a lower score, with a maximum score of 36 possible. Supporters showed a significant improvement in scores between baseline and 3 months (*P* < .001) and baseline to 6 months (*P* < .05).

##### Global Change Measure

Only three supporters completed the measure at the 3-month stage. They provided the same perspective as participants, in that overall participants’ wellbeing showed no change. Regarding participants’ independence, each of the three supporters selected a different response from ‘A bit more independent’, ‘No change’, and ‘Much less independent’.

At 6 months, few supporters completed the questions (n = 6). One reported that their relative’s wellbeing was much improved, two reported no change, two a bit worse, and one much worse. Again, supporters reported similar views to participants, with 66.7% (n = 4) identifying no change and the other two supporters saying their relatives had become less independent.

#### Qualitative Data Themes

Reflexive thematic analysis of the data led to the development of the following main themes: ‘positive validation for the PRIDE-app principle and concept’; ‘importance of facilitator’; and ‘recommendations to improve the PRIDE-app’. Each theme had several subthemes, and these, along with the main themes, often overlapped between the RE-AIM elements (Table 3). Participants living with dementia are labelled Pt, supporters as Sup, and facilitators as DA, each followed by their corresponding interviewee number.

**Table 3.** The Four Central Themes, the Sub-themes and the Relation to the RE-AIM Elements.

Themes	Sub-themes and their relation to RE-AIM framework			
	Reach	Effectiveness	Adoption	Implementation and Maintenance
T1: Positive validation for PRIDE-app principle and concept	❖ Motivation and engagement of users			
	❖ IT literacy and confidence		❖ IT literacy and confidence	
			❖ Continuation of PRIDE-app techniques	
		❖ Reflection		
T2: Readiness to face and attitudes towards dementia	❖ Reaction to and acceptance of diagnosis		❖ Reaction to and acceptance of diagnosis	
	❖ View of supporters on dementia and participant abilities		❖ View of supporters on dementia and participant abilities	
T3: User engagement with and the importance of the facilitator role		❖ Source of encouragement and reassurance		
		❖ Facilitators provide an independent voice		
		❖ Necessity of Dementia Advisors		❖ Necessity of Dementia Advisors
T4: Recommendations for improving the accessibility, usability and delivery of the PRIDE-app	❖ Dementia accessibility			
	❖ Format and delivery to meet user needs			
	❖ Ways to improve the usability of the app			
		❖ Navigation and signposting		

##### Theme One: Positive Validation for PRIDE-app Principle and ConceptMotivation and Engagement

There was a positive view of the overall PRIDE-app intervention and what it aimed to achieve. Motivation was an important factor in the decision to take part in the study and apply the techniques the PRIDE-app encouraged, therefore identifying a potential characteristic in people with dementia or their families that would make them a suitable user of the app:

*A lot of people wanted to do this because of the goal-setting and they wanted that aspect for their mum or partner or whatever. And I think there’s a lot to be said for giving that motivation and that kind of focus for people who potentially would just sit in a chair* (DA 3)

DAs reported positively on the influence of the intervention in encouraging their participants. Supporters appreciated the additional support the PRIDE-app content provided in areas where they were uncertain how to help their loved ones:

*He’s become so much more motivated just through those three sessions, and wanting to do things* (DA 3)

###### IT Literacy and Confidence

Not all participants felt they were the target group for the PRIDE-app intervention, with computer literacy linked to motivation to engage with the app. Similarly, DAs felt that although participants engaged with the sessions and wanted to use the app in their own time, some were wary of using it by themselves due to the online format:

*But looking at the app, it seemed fairly easy to follow, and I would suggest for those that were competent in computers, but…for us, unfortunately, it was just a step too far* (Sup 3)

*I felt that it was good whilst we were going through and getting to the goals. But I was very much aware that whilst there was an intention with some people, most of them didn’t actually go back in between* (DA 3)

Additionally, one participant was already using several techniques identified in the PRIDE-app to manage their dementia. Therefore, they felt less motivated to use it outside of the research environment.

###### Reflection and Continuation of Techniques

The PRIDE-app provided a useful stimulus for reflection, DAs worked with participants to tailor use of the app techniques to suit their lifestyles and different dementias:

*It can just be about confidence, not capability, so something like this [PRIDE*-*app] can really help that set of participants because it just builds on their autonomy and reminds them that they can do things and it sets them goals and all of that stuff that they can work towards* (DA 2)

A few participants were already focused on continuing the techniques that the PRIDE-app had encouraged, whether new or pre-existing. Two requested additional sessions with their DA because they were enjoying the goal-setting process. DAs were very positive about the PRIDE-app and believed it had a place within services to support people on clinical waiting lists:

*It could be really useful for things like cognitive stimulation groups, memory clinics…it would be useful to people on waiting lists for sure, setting goals, and things like that* (DA 2)

Facilitating the sessions prompted one DA to consider implementing the app’s goal-setting technique into their services:

*I mean, it’s given me the idea that I’d like to do some face-to-face groups that are all about goal-setting because of the ideas of people [PRIDE-app participants] are coming up with* (DA 3)

##### Theme Two: Readiness to Face Dementia

The readiness to accept their diagnosis and understand the potential loss of social identity appeared important factors in whether participants were able to engage as fully as intended with the app and have the level of insight required to make the behavioural changes PRIDE promoted. Participants displayed mixed reactions to their dementia diagnosis, with some acknowledging it and others distancing themselves. A sense of confusion about being diagnosed was evident in some:

*It hit me at the beginning about being told that I’ve got dementia…I thought that was a bit odd* (Pt 2)

Despite an official dementia diagnosis being the prerequisite criteria, one participant (Pt 1) wavered between acknowledging their diagnosis and distancing themselves from the wider dementia population:

*I keep saying “they”. I mean, maybe it’s me as well, I don’t know*

Participants expressed frustration about the impact of dementia on their independence, such as their ability to drive, and on their social role. Supporters voiced their personal views on their experience with dementia:

*[Person with dementia] been in denial for the last year or more, but the last few months, she had accepted having mild Alzheimer’s…she has done, to some extent, fundamentally, but perhaps not emotionally* (Sup 2)

##### Theme Three: User Engagement with and the Importance of the Facilitator Role

The role of the DAs was identified as a pivotal factor in how well people with dementia engaged with the PRIDE-app. Several key behaviours and skills were identified as the core components of an effective facilitator. The DAs provided an independent voice within the dementia dyad, a relationship which could often be strained by dementia:

*It is useful to have somebody like [Dementia Advisor] engaged with you, that third party is very, very valuable* (Sup 1)

From the perspectives of those living with dementia, they appreciated having the external voice of the DAs, who saw them as individuals and validated and respected them. DAs acted as an additional source of encouragement and reassurance for those with dementia and their supporters. One supporter emphasised how the positive, inquiring attitude of their facilitator was key in their participation:

*The way that [Dementia Advisor], as it were, inquired and encouraged [Person with dementia] was more helpful than could be achieved simply by reading out [the information] or assuming that’s good* (Sup 2)

The facilitators themselves felt that the PRIDE-app enabled discussions between Advisors and those with dementia, but also within the dyads:

*I think it’s also a good communication tool…for a carer to understand how that person’s feeling* (DA 2)

Supporters sought reassurance regarding their role and whether they were doing the right thing for their loved one with dementia. Advisors were seen as a source of knowledge and reassurance:

*We were doing these things before, but I didn’t realise perhaps until the PRIDE app and discussing with [Dementia Advisor], that this was the right thing to do, as it were, or that it was providing the sort of stimulus that I was seeking it provides* (Sup 2)

It was evident from the interviews how essential DAs were for the delivery of the PRIDE-app. Differing levels of computer literacy meant participants were reliant upon Advisors’ guidance when using the app. As a result, few were able or confident enough to use it independently:

*I think people are nervous about using an app…and this is using experience from other similar studies as well, is that they start off quite confident, and then…they press the wrong button or something…and then they won’t revisit it at all…it feeds into their feelings of incompetency* (DA 2)

The remote delivery of the intervention caused some communication difficulties between participants and DAs, and Advisors were uncertain whether they were delivering the app as intended:

*It’s very difficult to know when you’re giving a verbal instruction without any visual whether that’s actually happening in the way that’s intended* (DA 2)

##### Theme Four: Recommendations for Improving the Accessibility, Usability and Delivery of the PRIDE-app

All interviews discussed where improvements could be made to the PRIDE-app. All users often found navigating the app difficult to understand and felt that the signposting could have been clearer:

*The navigation was the biggest issue…You’re never quite sure where you were at any given time* (Sup 1)

Interviewees suggested how to improve the accessibility and ease of use of the PRIDE-app through a clearer mapping system of the content, adding reminders on pages to inform users which content section there are on, and revising the interface:

*Maybe sort of more clear mapping system just so to return to this, to return to that might have been helpful* (DA 3)

*Maybe set it out just as a calendar and each…you know, just your log, you know, you go into that calendar date, you pick the date. Bang! That’s my activity within the activity. You could put a daily plan type of thing and something like that* (Pt 1)

Some felt that the app was not as dementia friendly as it could have been and believed that the end users were not considered enough in the development process:

*It feels a little bit like homework…whereas if it had a sort of fun…a little bit of a game, brain train-y type of thing…that might help people engage with it and actually not feel like it’s a chore to fill out on their own* (DA 2)

Supporters and advisors shared the perspective that altering the angle of the PRIDE-app and including more ‘push’ technology, taking some of the responsibility away from the user to remember to use the app, would increase engagement in people with dementia:

*I think instead of using pull technology, i.e., you’re requiring the patient to go online and do something, you should be using push technology…If you’re pushing them reminders, I think it would help significantly* (Sup 1)

Ideas related to the format were well discussed across the interviews. Supporters and advisors suggested that an online, smartphone or paper version, with in-person or virtual contact, would be best suited going forward to account more for personal preferences. The remote delivery of the PRIDE-app posed difficulties in facilitating the sessions, as Advisors were unable to check what the person with dementia was doing on the app, and the support provided was restricted by the format:

*I couldn’t see what she was doing, I couldn’t see what she was inputting. I think that’s the drawback* (DA 2)

Advisors used their experience from similar studies to provide recommendations on how the delivery of the PRIDE-app could be improved going forward and have a positive impact on its adoption and maintenance by services and users:

*Before Covid…we’d have gone out and done a face-to-face visit with the person and maybe a carer. We would have taken them through the program, showing them what they have to do…okay, I’m going to call you next week, so, you can use it as much as you want in the time…and that would have raised their confidence…I might say let’s book in a weekly session where I call you and we go through the session together every time, not having them do it on their own in between because I’m pretty sure my lady didn’t do very much on her own* (DA 2)

## Discussion

This study was an innovative and successful use of the RE-AIM framework to evaluate the feasibility and effectiveness of the PRIDE-app. According to the outcome data, no improvements had been observed. Results for supporters were mixed, with better mental health but lower quality of life at follow-up. There was no change in daily activities ratings by participants, which was anticipated at the baseline measures, as the participants had mild dementia where the symptoms would not have impacted their daily activities too considerably. As the data did not show any real improvements in participants’ functional activities, independence or general wellbeing, this provided no indication that the PRIDE-app was effective. Table 4 outlines how the RE-AIM concepts were explored through analyses of quantitative and qualitative data.

**Table 4.** How Reach, Effectiveness, Adoption, Implementation, and Maintenance (RE-AIM^6^; Dimensions Were Addressed in the Study.

RE-AIM dimension	Definition	How Addressed in the Study	Findings
Reach	The absolute number, proportion, and representativeness of individuals contacted and those who are willing to participate in the intervention and reasons given as to why or why not choose to participate in the study.	Recruitment and characteristic figures; Level of participant engagement with the PRIDE^a^-app.	• All participants were White British
			• Prior tech experience in participants ranged from none to extensive
			• Motivation a key influence in people taking part and applying the techniques
Effectiveness	What are the outcomes associated with using the PRIDE-app and are there any potential negative effects?	Change of pre- and post-intervention scores: CASP-19^b^, IADL^c^, EQ-5D-5L^d^, GHQ-12^e^, GDS^f^, EID-Q^g^, and global change measure.	• No significant changes found across participant measures
			• A decline in supporter quality of life reported, but an improvement in their general health
Adoption	The absolute number, proportion, and representativeness of settings and the target patient group and intervention facilitators who are willing to initiate a program and why.	Post-intervention qualitative interviews with participants; app use; participant retention rate; interviews with facilitators.	• Retention of participants dropped between baseline and follow-ups
			• App not originally used as intended – users were able to modify to fit their lifestyles
			• Regular use not associated with how valued the content was by users
Implementation	The extent to which an intervention may be delivered as intended and whether individuals would use the intervention.	Interviews with participants and facilitators (information on delivery, barriers for delivery, and implementation)	• Continued use of techniques post-study by users and DAs
			• Tech experience and remote delivery potential barriers
			• Role of DAs pivotal in delivery
			• Participants would prefer a choice of formats in future
Maintenance	The long-term effects on outcomes (usually 6 or more months) and the extent a program becomes part of routine practice.	Interviews with participants and facilitators (whether the app would fit into the existing services)	• Stakeholders believed content was relevant and had a place among memory services

^a^PRIDE: Promoting Independence in Dementia.

^b^CASP-19: Control, Autonomy, Self-realization, and Pleasure Scale-19.

^c^IADL: Lawton Instrumental Activities of Daily Living Scale.

^d^EQ-5D-5 L: EuroQoL Quality of Life.

^e^GHQ-12: General Health Questionnaire – 12.

^f^GDS: Geriatric Depression Scale.

^g^EID-Q: Engagement and Independence in Dementia Questionnaire.

Participants and supporters emphasized the engagement and reflection the PRIDE-app encouraged as crucial factors in whether they felt they had benefited from the intervention. Motivation was a key factor in whether participants applied the techniques from the PRIDE-app, and goal-setting was a popular concept with participants and supporters. The PRIDE-app appeared useful in giving participants and supporters more information and guidance on adjusting to a diagnosis and providing techniques that some used effectively in their everyday lives. Supporters appreciated how the app contributed to their understanding of dementia and how they could best support their loved ones. The role of the DAs was a vital factor in how users engaged with and perceived the PRIDE-app. Their role provided an independent perspective and encouragement that enabled positive discussions between themselves and participants and within dyads. This indicates that establishing a personalized, trusting relationship between facilitator and service user is crucial for the successful implementation of the intervention. It was notable that DAs and supporters had more to say and seemed more enthusiastic about the PRIDE-app than participants.

The reach of the PRIDE-app intervention was limited regarding ethnicity, as all participants were white British. Although the data could not explain why this was, the inclusion of translated versions of PRIDE, and the involvement of more under-represented communities in the development and recruitment stages, may potentially bridge this gap. The data suggest that improvements to the PRIDE login system and navigation are needed to widen the user audience to include both those confident with computer technology and those who need more support. DAs were positive about the app and believed it could have a place in services, whereas supporters often thought it was too complex for their relatives. People with dementia felt that the app could be beneficial to them, but its current interface and usability made it appear more like homework, rather than an enjoyable resource. These perspectives should be considered when enabling access to the PRIDE-app, to ensure as wide a reach as possible and encourage less-confident individuals to try the app with additional support. With improvement, the reach of the PRIDE-app could be increased to include a more diverse and representative sample of the population of persons living with dementia, potentially enabling more people to benefit from its content and techniques.

Participants and supporters did not adopt the PRIDE-app in their daily lives as originally intended, as the online format was challenging for some users and deemed not very accessible. However, some did adopt the techniques promoted and they continued to apply these after the study, suggesting that the content was appropriate and relevant. Interviews demonstrated the importance of motivation, computer literacy, and encouragement in whether participants used the app. The modifications discussed would likely increase adoption within this sample, as they would likely improved accessibility, usability and longer-term engagement with the PRIDE-app. DAs delivered the intervention sessions as intended, and participants were receptive to the discussions. Although many had prior experience with technology, adoption was lower than expected. Some participants showed a preference for a paper-based format, and confidence with computers varied within the sample. Participants and supporters sometimes modified their use of the PRIDE-app to fit in with their lifestyle by adopting the techniques promoted, rather than using the app itself to record goals and plans. This reaffirms that the content was relevant and applicable to those living with mild dementia and that regular use was not necessarily required for the contents to be valuable.

As participants, supporters and facilitators continued to use the techniques promoted by PRIDE and aimed to incorporate them in other service areas, this suggests that the app and/or its principles might have a place within dementia support services. The key stakeholders agreed that the content is relevant. The DAs were keen on participants engaging with the PRIDE-app, and a positive working relationship was identified as a key component to implementation. For future use of the PRIDE-app in dementia services, facilitators would need to work collaboratively with users over a longer duration of sessions, to monitor and motivate continued use of the app and its techniques.

### Findings in Relation to Previous Work

Studies of web- and app-based interventions, such as the PRIDE-app, have reported similar findings in terms of usability and adoption. The ReACT app^17^ had a number of features to help people with dementia to structure their days, including a calendar, diary, checklists and personal contacts. In contrast to the PRIDE-app study, Øksneberg et al. found that the level of technological experience and support was a factor in whether or not participants adopted the ReACT app. However, similarities were found regarding caregiver involvement being a strong influence in whether participants adopted the ReACT app. Although the PRIDE-app covers a wider range of topics than the ReACT app, Øksneberg et al.’s work provides additional insight into technological interventions and the factors often affecting adoption in people living with dementia.

### Methodological Issues and Limitations

Due to the challenges of the COVID-19 pandemic, all interviews had to be conducted remotely, which meant that ARL was unable to fully grasp interviewees’ gestures or body language, especially during telephone interviews. This could have meant that some meaningful interpretation of the data was missed. However, as question schedules were provided prior to the interview itself, this gave people time to consider their answers and make notes of everything they wanted to say. As with all qualitative research, subjectivity and the interpretation of data are outstanding methodological limitations, as ARL created the interview schedules, conducted them and was lead on the analysis. The inclusion of a second reviewer aimed to limit the influence of any internal bias or views from ARL that could have affected the interpretation of data. As an experienced clinician and qualitative researcher, in particular dementia research, OM provided objective and professional insight across the data. The collaborative reflection during the thematic analysis process ensured that agreement was reached between both reviewers at the end of each stage.

All participants were of white British ethnicity, and interest from nonwhite communities was non-existent except for one volunteer on JDR. We could not demonstrate the reasons for this, but according to the existing evidence, cultural and religious differences have been identified in understanding dementia and accessing dementia support.^18,19^ Additionally, the potential ‘effectiveness’ of the PRIDE-app intervention was not fully measurable through this study due to the restrictions enforced by the COVID-19 pandemic lockdowns and their impact on the nature of the PRIDE interventions (e.g., promoting social health and psychological independence through expanding one’s social network) and data collected.

## Conclusion

Technological literacy appeared to be a barrier that prevented some participants and supporters from engaging fully with the PRIDE-app. It could be that people with young onset dementia would be more likely to adopt the PRIDE-app, as they would generally be more computer literate than older adults. Many interviewees thought the app was a good idea but was too complex to engage with fully. The choice of an online, smartphone app or paper-based version of the PRIDE-app in the future would likely increase the reach and adoption of the intervention. Although most participants did not take full advantage of the PRIDE-app and incorporate it into their daily activities, the interviews suggested potential benefits across participants, supporters and facilitators.

Even though the adoption of the PRIDE-app by participants was not as originally intended, the findings from fidelity checklists and interviews indicated the important role of motivation, encouragement, and a good level of computer literacy in determining adoption of the app. Qualitative data identified motivation, together with an understanding and acceptance of their dementia diagnosis, as key components necessary for the target user to fully engage with the app. Interviewees’ recommendations for improving accessibility and engagement with the PRIDE-app should be considered to increase its adoption in the future. The views of supporters should also be considered when offering the PRIDE-app intervention, as their strong personal views on dementia and what people are capable of could potentially limit access to the app:

There were reports of participants and facilitators continuing to use the PRIDE techniques and apply them further in other service areas after the study. This suggests that the PRIDE-app has a place within dementia services and that the content resonates with people living with dementia. Interviews demonstrated the necessity for a DA who could work collaboratively and positively with participants. This important factor should be considered if implementing the PRIDE-app in future services. Enabling long-term implementation and maintenance may require offering people with dementia alternative formats of the app, alongside the original online version, and involving face-to-face elements during the intervention delivery.

The perspective of dementia accessibility is pivotal in ensuring the PRIDE-app is useable and accessible for the target audience. The quotes gathered do portray some of the stigma which goes with dementia-specific technology, as individuals may not want to be seen using dementia-specific technology. Addressing and incorporating the different perspectives of key stakeholders would significantly improve the chances of the app being adopted and implemented by healthcare services and people living with dementia.

## Appendix

### Abbreviations

ARL: Dr Abigail Rebecca Lee
CASP-19: Control, Autonomy, Self-realization, and Pleasure Scale-19
CRN: Clinical Research Network
DA: Dementia Advisor
EID-Q: Engagement and Independence in Dementia Questionnaire.
GDS: Geriatric Depression Scale
GHQ-12: General Health questionnaire – 12
IADL: Lawton Instrumental Activities of Daily Living Scale
JDR: Join Dementia Research
OM: Dr Orii McDermott
PRIDE: Promoting Independence in Dementia
RE-AIM: Reach, Effectiveness, Adoption, Implementation and Maintenance framework
